# 

**Published:** 1894-03-31

**Authors:** 


**Notice to Advertisers and Subscribers.**

All Advertisements, Orders for Copies of The Hospital, and Business Communications should be addressed to **The Publisher, NOT TO THE EDITOR**, The Hospital, 428, Strand, W.C.

The Cost of Subscription, post free, is as follows:—Per week, 2½d.; per quarter, 2s. 8d.; per half-year, 5s. 3d.; per annum, 10s. 6d. Foreign:—Per Annum, 12s. 8d.; payable, in all cases, in advance.

**OUR NEW OFFICES.**

428, Strand, W.C., will henceforth be the Office of this Journal.

**Notices of Births, Deaths, and Marriages** are inserted in this column at a charge of 2s. 6d. for an announcement not exceeding four lines.

### NOTICE TO CORRESPONDENTS.

All MS., Letters, Books for Review, and other matters intended for the Editor should be addressed The Editor, The Lodge, Porchester Square, London, W.

The Editor cannot undertake to return rejected MS., even when accompanied by stamped directed envelope.

**"Burdett's Hospital Annual," 1894.**

Fifth year of publication. 900 pp. Boards, gilt lettering. A most complete guide to British, Colonial, Indian, and American Hospitals, Dispensaries, Nursing and Convalescent Institutions, and Asylums. Price 5s. Published by The Scientific Press (Limited), 428, Strand, W.C.

**NOTICE.—The Index for Vol. XIV. (ending Sept. 30th, 1893) is on sale at the Office of this Journal, price 2½d., post free.**

### SCRAPS AND GLEANINGS.

Mr. Ludwig Mond has again sent a donation of £100 to the Hospital Sunday Fund.

A legacy of £5,000 has been bequeathed by the late Mr. W. T. Whitaker to the Hospital Sunday Fund.

Baron Hirsch has sent a further donation of £600 to the funds of the Brompton Hospital for Consumption.

The Lord Mayor will preside at a festival dinner on behalf of the funds of the Metropolitan Hospital on April 30th.

At a special meeting of the Woking Local Board it was agreed to co-operate with Guildford in the matter of an isolation hospital.

The Oldham Infirmary showed at the annual audit of accounts that the current income had been about equal to the current outlay.

The Government has signalised an intention of contributing £15,000 towards the establishment of a veterinary college for Ireland. The site will be selected in Dublin.

The Torbay Hospital is about to open an ophthalmic department in connection with the institution and the appointment of honorary ophthalmic surgeon will be made next month.

The Great Northern Central Hospital has received a further donation of £300 from the Baron de Hirsch towards the £5,000 still required to pay off the debt on the new buildings.

At the annual meeting of the Blackheath and Charlton Cottage Hospital regrets were expressed that the committee were unable to show a balance of receipts over expenditure.

The St. John Ambulance Association has forwarded, through its Capetown centre, a supply of stores to Buluwayo, where ambulance classes are being formed for the police force stationed there.

Mr. William Garton, of Woolston, Southampton, has presented £500 to the Council of the Hartley Institution towards the cost of the new engineering laboratory which is about to be added to that institution.

Upon the recommendation of the medical staff, a bath-room has been constructed at the Norwich Eye Infirmary with all the necessary conveniences, and this has proved a source of comfort and advantage to the patients.

A welcome addition of £200 to the "Endowment Fund" of the Princess Alice Memorial Hospital, Eastbourne, is announced to have been received from "A Friend," during the period under review at the last annual meeting

The Frome Cottage Hospital presented a very creditable balance-sheet at the recent annual meeting. The year under review commenced with a balance in hand of £19, which, at the end, and after payment of all expenses, increased to £51.

A donation of £5,000 has been made to the New Infirmary, Halifax, by Miss Rawson, of Millhouse. This is for the purpose of erecting a new ward to perpetuate the memory of Mr. William Henry Rawson, who had been a benefactor to the institution.

The in-patients of the Victoria Hospital, Folkestone, have contributed £50 towards the debt of £300 at present existing on the hospital accounts. A large local bazaar is organised to take place during this month, in favour of the finances of the institution.

The report of the Newbury District Hospital is a satisfactory one. The bui ding affords accommodation for twelve patients, and it has been practically full all the year round; and while the number of patients has been greater the average cost has been less.

Mr. Charles Scotter, general manager of the London and South-Western Railway, has presented the sum of £500 to the South-Western Orphanage in dedication of a cot in remembrance of the late Mrs. Scotter, and her constant and generous sympathy with the institute.

The Earl of Derby has consented to lay the foundation-stone of the new hospital at Fleetwood on April 7th. The late earl opened the larger wing of the institution ten years ago. A legacy of £1,000 has been received from the late Miss Henderson towards the funds of this hospital.

The committee of the Yeatman Hospital, Sherborne, has issued a special appeal asking for subscriptions to meet the outlay incurred on the alterations recently executed on the building; the amount realised by this appeal has been £283, of which the Rev. O. A. Benthall contributed £100.

The governors of the New Hospital for Consumption, which it has been determined to establish in Ireland, have selected a site near Newcastle, Co. Wicklow. There are to be eight residential blocks, but at present only two will be erected. The cost of the whole is estimated at £9,000.

The Hunstanton Convalescent Home is extending its premises, and the committee have purchased two houses in the rear of the establishment, which extra accommodation has proved itself a necessity, since the number of applications to the Home have greatly increased during the past year.

A large bazaar is to be held in August in furtherance of the finances for the Oban Cottage Hospital. The name of H.R.H. Princess Louise heads the list of patronesses. The sum of £1,300 has already been subscribed towards the scheme for the building of this much-required institution.

The contributions towards the Glasgow Lock Hospital show a falling off of £22, as compared with the previous year's; the directors are appealing for further contributions to carry on the work of the institution. This hospital is doing an excellent work in a quiet and unobtrusive manner.

It was resolved last year to change the termination of the financial year of the Ross Memorial Hospital from July 31st to December 31st, so that, in the annual meeting of subscribers recently held, the period under review was seventeen instead of twelve months. The finances are in a satisfactory condition, there being a balance of £350 to the good.

The treasurer of the Royal Victoria Hospital, Bournemouth, has received £287 towards the support of the institution from Mrs. Henry Newlyn, by whose efforts the sum was raised in subscription. During the year, 3,659 cases have received treatment at this hospital. The annual subscriptions towards this charity, though they show an improvement on the previous year, still are reported to remain very unsatisfactory.

### Medical Vacancies and Appointments

### APPOINTMENTS.

**E. France, M.B., B.S.Dunelm, appointed House Surgeon to the Belgrave Hospital for Children, S.W. E. K. Houchin, L.R.C.P. and S.Edin., appointed Deputy Coroner for the Eastern Division of London. J. H. Morgan, M.A., F.R.C.S., appointed Special Lecturer on Clinical Surgery to Charing Cross Hospital Medical School. J. M. S. Preston, M.B., C.M.Edin., appointed Medical Officer for the Third District of the Township of Manchester. W. P. Purvis, M.R.C.S.Eng., appointed House Surgeon to the Royal South Hants Infirmary. H.S.Ware, B.A., M.B., B.S.Camb., appointed Resident Medical Officer to the City of London Hospital for Diseases of the Chest, Victoria Park. C. E. Woakes, M.R.C.S., L.R.C.P.Lond., appointed House Surgeon to the London Throat Hospital, Great Portland Street, W.**

### VACANCIES.

#### Resident Medical Officers.

Birmingham and Midland Free Hospital for Sick Children.—A Resident Medical Officer and a Resident Surgical Officer. Salaries £70 and £50 respectively, with board, washing, and attendance. Applications to the Secretary, Children's Hospital, Steelhouse Lane, Birmingham, by April 4th.

Carnarvonshire and Anglesey Infirmary, Bangor.—House Surgeon. A knowledge of the Welsh language is desirable. Salary £70 per annum, with board and lodging in the house. Applications and testimonials to the Secretary by April 7th.

East London Hospital for Children, Glamis Road, Shadwell, E.—House Physician. No salary, but board, lodging, and washing are provided. Applications and testimonials to Thomas Hayes, Secretary, by April 4th.

General Hospital, Birmingham.—Two Assistant House Surgeons. Appointments for six months, and may be held by re-election for a further period of three or six months, but no longer. No salary attached to the posts, but residence, board, and washing will be provided. Applications and testimonials to Howard J. Collins, House Governor.

Leith Hospital.—House Physician, House Surgeon, and Surgeon for the Outdoor Department. Appointments for six months. Salary to each office £50 a year, with board in the hospital. Applications to George Mann, Secretary, 33, Bernard Street, Leith, by April 4th.

Liverpool Eye and Ear Infirmary.—House Surgeon, doubly qualified. Salary £80 per annum, with residence and maintenance. Applications to Reginald Haigh, Honorary Secretary, 13, Bevey's Buildings, George Street, Liverpool, by April 7th.

Morpeth Dispensary.—House Surgeon, doubly qualified, unmarried. Salary £120 per annum, with furnished house, coals, and gas. Applications to the Honorary Secretary, N. J. Wright, Morpeth, Northumberland, by April 4th.

National Hospital for Diseases of the Heart and Paralysis, 33, Soho Square, W.—Resident Medical Officer, doubly qualified. Appointment for six months. Honorarium 10 guineas. Applications to the Secretary.

#### Non-Resident Medical Officers.

City of London Hospital for Diseases of the Chest, Victoria Park, E.—Pathologist. Salary, 100 guineas per annum. Applications to the Secretary at the office, 24, Finsbury Circus, E C., by April 12th.

County of Northumberland.—Medical Officer of Health. Salary £500 per annum, with travelling expenses. Appointment for three years. Applications and testimonials, endorsed "Medical Officer," to C. D. Forster, Clerk to the Council, by March 24th.

Owens College, Manchester.—Professor of Zoology. Applications to the Council of the College, under cover to the Registrar, by April 3rd.

Royal College of Surgeons in Ireland.—Secretary of Council; must be a Fellow of the College. Particulars as to salary and duties from Mr. G. F. Blake, Registrar, at the College.

Sheffield School of Medicine, Leopold Street, Sheffield.—Tutor to take charge of Dissecting Room, and hold classes in Anatomy and Physiology. Salary £125 per annum. Applications to the Secretary by April 2nd.

South-Eastern Fever Hospital, Hatfield Street, New Cross Road, S.E.—Dispenser, qualified under the Pharmacy Act. Salary 35s. per week and dinner daily. Applications to the Medical Superintendent.

University College, Bristol.—Medical Tutor. Salary £125 per annum. Applications and testimonials to E. Markham Skerritt, Dean, by April 4th.

### The Hospital Advertiser.

### BOOKS FOR NURSES.

Demy 8vo., 200 pp., cloth extra, price 3s. 6d.,

**SURGICAL WARD-WORK AND NURSING**: A practical Manual of Clinical Instruction for Nurses and Students. This book will be found especially useful to beginners, to whom its simple description of elementary principles and treatment in surgery will render it invaluable. By ALEXANDER MILES, M.D. (Edin.), C.M., F.R.C.S.E. Over one hundred illustrations.

LIST OF CHAPTERS.

SECTION I.—ANTISEPTIC SURGERY. Chap. 1. General Principles of Antiseptic Surgery—2. Antiseptic Lotions—3. Antiseptic Powders and Unguents—4. Materials for Dressings—5. Lotion Basins—6. Ward Table and Dressing Tray—7. Antiseptic Dressing—8. A Spray Dressing—9. Management of Surgical operations—10. The Lotion Table—11. Dressings Table—Anæsthetics—12. Preparation of Patient for Operation—13. Treatment after Operation—14. Nursing of Special Cases after Operation.

SECTION II.—THE USE OF REST IN SURGERY. 15. Extension Apparatus—16. Plaster and Starch Cases—17. Appliances for Joint Affections—18. Appliances for Fractured Bones.

SECTION III.—BANDAGING. 19. Bandaging—20. Special Bandages—21. Special Bandages (*continued*)—22. Sspecial Bandages (*continued*).

SECTION IV.—SURGICAL INSTRUMENTS AND APPLIANCES. 23. General Instruments—24. Instruments for Hæmorrhage—25. Knives—26. Saws—27. Bone Instruments—28. Instruments used in Trephining—29. Mouth Instruments, etc.—30. Intestinal Instruments—31. Urethral Instruments—32. Lithotomy and Lithotrity Instruments—33. Laryngeal Instruments—34. Aural, Nasal, and Ophthalmic Instruments—35. Gynæcological Instruments.

"Will furnish much-needed guidance to the student and the nurse in the early stages of their apprenticeship."—*Times*.

"This is a fully illustrated handbook for the use of junior students of medicine and nurses, which should prove of very great value, Dr. Miles being unquestionably an authority on the subject of which he writes."—*Publishers' Circular*.

Just ready, Important New Text-Book of Ophthalmic Nursing.

Crown 8vo., cloth gilt, pp. 200, profusely illustrated, and with many woodcuts, price 3s. 6d., post free,

**OPHTHALMIC NURSING.** By SYDNEY STEPHENSON, M.B., F.R.C.S.E., Surgeon to the Ophthalmic School, Hanwell, W., &c., &c. A complete text-book on this branch of nursing, illustrated by numbers of original figures, and containing a glossary of terms and list and description of instruments used in ophthalmic nursing.

As yet no book has been published dealing with nursing in diseases of the eye. As a text-book, "Ophthalmic Nursing" will be invaluable to nurses desiring to qualify in this particular branch of nursing. The managers of Ophthalmic Institutions are invited to adopt this work as a text-book for their nursing staffs. The book contains a frontispiece, "Vertical Section of the Eye-ball and Eye-lids," and sixty-one other illustrations It is concisely yet exhaustively written, and its method of treatment and style will render it especially serviceable to the Nursing Profession.

THE LANCET says: "The work now before us gives the result of his experience to those who have charge of Ophthalmic patients, and will prove useful. A good account is given of the proper arrangements and preparations that should be made for operations, with the various modes of bandaging one or both eyes. The work may very advantageously be studied by nurses and clinical clerks, or dressers who are about to enter the Ophthalmic service of a hospital."

NURSING NOTES says: "A book on Ophthalmic Nursing has been a real need which is now well supplied. . . . . Those contemplating going in for Ophthalmic Nursing should study this useful work. . . . . One of the most useful books for nurses we have seen for a long time."

THE OPHTHALMIC REVIEW says: "The author has evidently endeavoured to write in language which will be intelligible to nurses of all grades, and we congratulate him on the clearness with which his statements are expressed. . . . . Chapters II. and III., upon 'The Germ Theory of Disease' and 'Contagion and Infection,' are good, and the latter contains useful rules for the avoidance by nurses and others of the contagion of eye disease."

"Will prove of inestimable value."—*Publishers' Circular*.

Third Edition, enlarged and partly re-written, cloth gilt, crown 8vo., about 200 pp., price 2s. 6d.,

**HOSPITAL SISTERS AND THEIR DUTIES.** By EVA C. E. LÜCKES, Matron to the London Hospital. Every matron, sister, or nurse who aspires to become a sister should read the valuable advice and information contained in this book. The author's experience as matron of London's largest hospital peculiarly qualifies her to speak on this subject.

"We cordially recommend the work to all hospital authorities as well as to the class for whose special benefit it is more immediately intended."—*Daily Chronicle*. "On every page we find evidence of Miss Lückes' thorough acquaintance with all the details of the nursing department of hospital management."—*Lancet*. "It is well worthy of study for those to whom it is addressed, and for all who are interested in the matter of nursing."—*New York Medical Journal*.

Second Edition, enlarged, demy 8vo., profusely illustrated, nearly 200 pp., cloth. Important Text-Book for Asylum Attendants, price 2s. 6d., post free,

**MENTAL NURSING.** A Text-Book specially designed for the instruction of Attendants on the Insane. By WILLIAM HARDING, M.B.

"Nothing could be better devised to serve as a text-book. The lectures are so simple and so instructive that they cannot fail to impress and interest readers. . . . Of great value to all nurses. . . . No institution should fail to supply their officials with a copy of the work."—*Local Government Journal*. "The book is written in a clear, easily understood style."—*Glasgow Medical Journal*.

Demy 8vo., 200 pp., Fourth Thousand, profusely illustrated with Copyright Portraits and Drawings, price 2s. 6d., post free,

**HOW TO BECOME A NURSE: And How to Succeed.** Compiled by HONNOR MORTEN, Author of "Sketches of Hospital Life," "The Nurses' Dictionary," &c. A complete collection of information of every kind such as persons desiring to enter nursing can want to know. General advice, useful hints, and full lists of hospitals, nursing schools, and other institutions to which intending nurses can apply for admission as probationers. Also terms, fees, and every other detail.

"Thoroughly accurate and reliable."—*Young Woman*.

"Those wishing to become nurses will find much excellent advice and information of every description pleasantly given. The numerous illustrations make the book very attractive, it is well printed, and the contents so admirably arranged that inquirers can easily find the information they are in want of."—*Nursing Notes*.

"No better book could be put into the hands of a young girl desirous of devoting herself to this particular profession."—*Sala's Journal*.

"A book that I can most cordially recommend."—*Woman*.

"Very excellent guide to the profession."—*Gentlewoman*.

**London: THE SCIENTIFIC PRESS (Limited), 428, Strand, W.C.**

### IMPORTANT WORK ON HOSPITALS, &c.

Next Week. Royal 8vo., 500 pp., 60 illustrations, cloth gilt. Price 21s., net.

### HOSPITALS, DISPENSARIES, AND NURSING.

Being Transactions of Section III. International Congress of Charities, Correction, and Philanthrophy, held in Chicago, 1893.

This valuable collection of papers will be published in one handsome volume as above, and comprises a variety of subjects, each of which is dealt with by a representative and leading authority.

The edition will be limited, and the pages are not stereotyped.

As sole English publishers of the work, The Scientific Press are now booking subscriptions for early copies, which will be delivered on the day of publication.

### THE CONTENTS ARE AS FOLLOWS:—

The Principles of Nurse Training. Miss Florence Nightingale, London.
Nursing Work of the Religious Orders of the Roman Catholic Church. Cardinal Gibbons, Baltimore.
The Medical Charities of the English Metropolis. Lord Cathcart, London.
Hospitals in Relation to Public Health. Dr. J. S. Billings, U.S.A., Washington.
Military Hospitals. Dr. C. Grossheim, Surgeon-General, German Army, Berlin.
Hospital Finances. Henry C. Burdett, London.
Applicability of Hygiene to Modern Warfare. Lt.-Col. J. L. Notter, British Army, Netley, Eng.
Standards of Education for Nurses. Miss Isabel A. Hampton, Baltimore.
The Trustee of the Hospital. Richard Wood, Philadelphia.
Relation of Training Schools to Hospitals. Miss L. L. Dock, Chicago.
Relation of Medical Staff to Governing Bodies in Hospitals. Dr. Edward Cowles, Boston.
Hospital Administration. Dr. H. Mercke, Berlin.
Relation of Hospitals to Medical Education. Dr. Henry M. Hurd, Baltimore.
Hospital Accounts and Methods of Book-keeping. James R. Lathrop, New York.
Paying Patients in Hospitals. Dr. H. M. Lyman, Chicago.
Paris Free and Paying Hospitals. Drs. Alan Herbert and W. Douglas-Hogg, Paris.
Dispensaries. C. C. Savage, New York.
Utility, Peculiarities, and Special Needs of Hospitals for Children. Dr. W. W. Ord, London.
Naval Hospitals (20 illustrations). Dr. J. D. Gatewood, U.S.N.
Detention Hospitals for Insane and Alcoholic Cases. Dr. M. D. Field, New York.
Cottage Hospitals. Francis Vacher, Birkenhead, Eng.
Obstetric Hospitals (1 illustration). Dr. B. C. Hirst, Philadelphia.
Hospitals for Infectious Diseases. Dr. C. F. M. Pistor, Berlin.
Isolating Wards and Infectious Hospitals. Dr. G. H. M. Rowe, Boston.
Hospital for Contagious and Infectious Diseases (4 illustrations). Dr. M. L. Davis, Lancaster, Pa.
Isolation Wards and Hospitals for Contagious Diseases in Paris. Drs. A. Herbert and W. Douglas-Hogg, Paris.
Training Schools for Nurses in Paris. Dr. Leon La Forte, Paris.
Hospitals and Nursing in Amsterdam. Dr. Edward Stumpff, Amsterdam.
Nurses Homes. Miss L. L. Lett, Chicago.
Diet Kitchens in Hospitals. Dr. B. H. Stehman, Chicago.
Hospital Dietaries. Miss M. A. Boland, Baltimore.
Laundry of the University of Pennsylvania Hospital (1 illustration). Dr. A. C. Abbott, Philadelphia.
First Help in Hemorrhage. Prof. von Esmarch, Kiel, Germany.
First Aid to the Injured and How it Should be Taught. Dr. H. G. Beyer, U.S.N.
First Aid to the Injured from the Army Standpoint. Dr. Chas. Smart, U.S.A.
Organisation of First Aid to the Wounded in Paris. Drs. A. Herbert and W. Douglas-Hogg, Paris.
The Ambulance System of New York. George P. Ludlam, New York.
An Easy Method of Bedmaking and Improved Stretcher for Hospital and Military Use (1 illustration). Dr. E. D. Worthington, Sherbrooke, P.Q.
Hospital Saturday and Sunday. Frederick F. Cook, New York.
Training Schools in Great Britain. Miss A. C. Gibson, Scotland.
Trained Nursing in Berlin. Fraulein Louise Fuhrmann, Germany.
The Work of Deaconesses in Germany.
Training Schools in America. Miss Irene Sutliffe, New York.
Proper Organisation of Training Schools in America. Miss Louise Darche, New York.
Nurses as Heads of Hospitals. Miss E. P. Davis, Philadelphia.
Needs for an American Nurses' Association. Miss Edith Draper, Chicago.
The Royal National Pension Fund for Nurses. Miss Gordon, London.
District Nursing in England. Mrs. Dacre Craven, London.
The Origin and Present Work of Queen Victoria's Jubilee Institute for Nurses. Miss A. Hughes, London.
District Nursing in America. Miss C. E. Somerville, Lawrence, Mass.
Children's Hospitals. Miss Rogers, Washington.
The Nursing of the Insane. Miss May, Rochester, N.Y.
Workhouse Nurses' Association. Miss Louise Twining, Liverpool.
Obstetric Nursing. Miss G. Pope, Washington.
Midwifery as a Profession for Women. Mrs. Z. P. Smith, London.
Nursing in Homes, Private Hospitals and Sanitariums. Mrs. S. M. Baker, Battle Creek, Mich.
London Hospital Nurses' Home. Miss Eva C. E. Lückes, London.
Association for the Training of Attendants. Mrs. D. H. Kinney, Boston.
Red Cross and First Aid Societies. John Furley, London.
Description of the Montreal General Hospital (3 illustrations). Dr. W. F. Hamilton, Montreal.
Description of Royal Victoria Hospital (2 illustrations). Jno. J. Robson, Montreal.
Description of the Roosevelt Hospital (11 illustrations). James R. Lathrop, New York.
Description of the Johns Hopkins Hospital (19 illustrations). Dr. H. M. Hurd, Baltimore.

London: The Scientific Press (Limited), 428, Strand, W.C.

### ACCOUNT BOOKS FOR INSTITUTIONS.

Designed by HENRY C. BURDETT.

Ruled in accordance with the uniform system of accounts advocated by the Metropolitan Hospital Sunday Fund, and prepared for the convenience and assistance of Secretaries who present annual returns for participation in the Hospital Sunday Fund Grants.

These Books are ruled so that the various descriptions of Receipts and Expenditure, &c., may be entered uniformly under the special headings and in the columns prepared for them.

There are TWO SERIES, No. 1 being for the larger Institutions, and No. 2 for Cottage Hospitals and Small Institutions. The sets are sold complete, at a specially reduced price; or each book may be had separately, at the prices set out below:—

### SERIES No. 1.—FOR HOSPITALS AND INSTITUTIONS.

(Any of these Books may be purchased separately; a reduction is made if the complete set is taken.)

1. ANALYSIS JOURNAL. Royal, 350 pp. Divided into seven sections, with parchment tags. Headings as follows:—
   - A. MAINTENANCE.
     1. Provisions.
     2. Surgery & Dispensary.
     3. Domestic.
     4. Establishment Charges.
     5. Rent.
     6. Salaries.
     7. Miscellaneous Expenses
   - B. ADMINISTRATATION.
     1. Management.
     2. Finance.

   (Uniform with Headings in Income and Expenditure Table.) Each Heading is ruled with pages containing 15 columns. Price £3.
2. CASH BOOK. Foolscap, 300 pp., printed Headings, specially ruled. Price £1.
3. CASH ANALYSIS AND RECEIPT BOOK. Foolscap, extra width, 300 pp., ruled, with 15 columns, printed Headings identical with Income and Expenditure Table. Price £1 5s.
4. SECRETARY'S PETTY CASH BOOK. 300 pp., foolscap, ruled with 13 columns, printed Headings identical with Income and Expenditure Table. Price £1.
5. LIST OR REGISTER OF ANNUAL SUBSCRIBERS. Double foolscap, 300 pp., ruled with 10 columns, printed Headings for the years 1893-1900 inclusive. Price £1 15s.
6. ALPHABETICAL REGISTER BOOK. Foolscap, 300 pp., ruled, with date, name and address, and cash colums, cut into alphabetical sections, with totals at end for annual balances. Price £1 6s.
7. SUBSCRIPTION REGISTER. Foolscap, ruled with 8 columns, printed Headings for date when subscriptions become due, name and address, and cash columns for years 1893 to 1898 inclusive. Price £1 1s.
8. LINEN REGISTER. Foolscap long quarto, 300 pp., ruled with columns for Stock, Condemned, Remaining, and Issued Linen, also total in Ward and Remarks, also printed lists of Hospital Linen. Price 8s. 6d.
9. INCOME AND EXPENDITURE TABLE. Basis of uniform system. For yearly balances and statements. Royal. Price 6d. per sheet, or 5s. per dozen.
10. SPECIAL APPEAL ACCOUNT TABLE. Foolscap. Price 3d. per sheet, or 2s. 6d. per dozen.

PRICE OF SET COMPLETE, £10 NET.

### SERIES No. 2.—FOR COTTAGE HOSPITALS AND SMALLER INSTITUTIONS.

1. CASH ANALYSIS, RECEIPT, AND EXPENDITURE BOOK. Double foolscap, 300 pp., ruled with 18 columns, printed Headings identical with Income and Expenditure Table, specially prepared for Cottage Hospitals and Small Institutions. Price £1 15s.
2. SECRETARY'S PETTY CASH BOOK.—300 pp., foolscap, ruled with 13 columns, printed headings identical with Income and Expenditure Table. Price £1.
3. INCOME AND EXPENDITURE TABLE. Basis of uniform system. For yearly balances and statements. Royal. Price 6d. per sheet, or 5s. per dozen.
4. LINEN REGISTER. Foolscap, 300 pp., ruled with columns for Stock, Condemned, Remaining, and Issued Linen, also total in Ward and Remarks, also printed lists of Hospital Linen. Price 8s. 6d.
5. LIST OR REGISTER OF ANNUAL SUBSCRIBERS. Foolscap. 300 pp., ruled with columns for the years 1893-1900 inclusive. Price £1.
6. SPECIAL APPEAL ACCOUNT TABLES. Foolscap. Price 3d. per sheet, or 2s. 6d. per dozen.

PRICE OF SET COMPLETE, £4 NET.

The books are all uniformly bound in half basil, with gilt letterings, and are ruled on best paper, and in every way prepared for practical use at the offices of Institutions.

### ACCOUNTS, AUDIT, AND TENDERS; the Uniform System of,

For Hospitals and Institutions, with Certain Checks upon Expenditure. Also the Index of Classification. Compiled by a Committee of Hospital Secretaries, and adopted by a General Meeting of the same, 18th January, 1892. And certain Tender and other Forms for securing Economy. By HENRY C. BURDETT. Demy 8vo, cloth, 6s.

This is a hand-book to the account books, explaining their nature and uses, the method of keeping them, and the general principles upon which they were designed.

All having to do with hospital accounts will find much valuable information in this work.

Secretaries making up returns for participation in the Donations of the Metropolitan Hospital Sunday Fund will find it invaluable. It will be of special interest, also, to the officials of all Institutions—Treasurers, Secretaries, and Auditors—as it expounds practically the Uniform System, which is now being widely adopted as a means of saving labour and of bringing all systems of accounts into uniformity.

"This useful and practical little book. No one interested in the hospital question can treat with indifference anything which falls from Mr. Burdett. . . . . A uniform system of keeping their accounts. . . . Would be a boon to hospitals generally."—*British Medical Journal.*

London: THE SCIENTIFIC PRESS (Limited), 428, Strand, W.C.

